# Structural basis to stabilize the domain motion of BARD1-ARD BRCT by CstF50

**DOI:** 10.1038/s41598-017-03816-4

**Published:** 2017-06-20

**Authors:** Rajan Kumar Choudhary, Mohd Quadir Siddiqui, Pankaj S. Thapa, Nikhil Gadewal, Senthil Kumar Nachimuthu, Ashok K. Varma

**Affiliations:** 1 Advanced Centre for Treatment, Research and Education in Cancer, Kharghar, Navi Mumbai, Maharashtra, 410 210 India; 2 Homi Bhabha National Institute, Training School Complex, Anushaktinagar, Mumbai, 400 094 India; 3 Department of Biotechnology, Mizoram University (A Central University) Aizawl, 796 004 Mizoram, India

## Abstract

BRCA1 associated ring domain protein 1(BARD1) is a tumor suppressor protein having a wide role in cellular processes like cell-cycle checkpoint, DNA damage repair and maintenance of genomic integrity. Germ-line mutation Gln 564 His discovered in linker region of BARD1 leads to loss of binding to Cleavage stimulating factor (CstF50), which in turn instigates the premature mRNA transcript formation and apoptosis. We have studied the dynamics of ARD domain present in the BARD1 *wild-type* and mutant protein in association with CstF50 using biophysical, biochemical and molecular dynamics simulations. It has been observed that the ARD domain is relatively more flexible than the BRCT domain of BARD1. Further relative orientations of both the ARD and BRCT domains varies due to the highly flexible nature of the connecting linker region present between the domains. It has been observed that mutant ARD domain is more dynamic in nature compared to *wild-type* protein. Molecular docking studies between BARD1 Gln 564 His mutant and CstF50 shows the loss of interactions. Furthermore, domain motion of ARD present in BARD1 was stabilized when complexed with CstF50.

## Introduction

BRCA1 associated ring domain 1 (BARD1) is a binding partner of breast cancer associated protein (BRCA1)^1, 2^. BARD1 comprises different domains including N-terminus ring domain, an ankyrin repeat domain (ARD) and two-tandem BRCT domains^3^. BARD1-BRCA1, RING-RING domain complex is an E3 ubiquitin ligase complex which is important for many cellular processes like DNA damage repair, cell cycle checkpoint and genomic integrity^1, 4–8^. Mutations associated to breast and ovarian tumors present in the ring domain of BRCA1 impairs the protein-protein interactions and complex formation with BARD1^6, 9, 10^. ARD and BRCT domains are connected via 14 residue linker region and provide binding site for CstF50^11, 12^. The BARD1 ARD-BRCT domain is essentially required for homology-directed DNA (HDR) repair, as cells transfected with truncated BARD1 are unable to carry out HDR^13^. BRCA1–BARD1 complex with Cleavage stimulating factor (CstF50) has pivotal role in transcription-coupled DNA-damage repair (TCR)^11, 12^. CstF50 has 7 Tryptophan-Aspartate (WD-40) repeats that interacts to RNA polymerase II (RNAP II) through its N-terminal region^14, 15^ whereas BARD1 interacts through its 7^th^ WD-40 repeat^16, 17^. BARD1/BRCA1-CstF50 complex, after UV exposure leads to RNAP II CTD degradation^17–19^ and inhibition of 3’end- mRNA processing^16, 17, 20^. Missense mutations in the BARD1 have been reported in sporadic cases of ovarian, uterine, and breast carcinoma as well as in patients with familial breast or ovarian cancer^21–25^. Germ line mutation Gln 564 His reported in BARD1 disrupts the interactions with CstF50 and p53. BARD1 Gln 564 His mutation affects BARD1 mediated p53-dependent apoptosis and 3’- mRNA processing^12, 21, 26^.

Multidisciplinary approaches were explored to study the effect of Gln 564 His mutation on the stability of BARD1 ARD-BRCT domain and interactions with CstF50. Furthermore, protein-protein docking and molecular dynamics simulation approach were employed to unravel the effect of mutation on domain dynamics of BARD1 ARD-BRCT, Gln 564 His mutant and BARD1 ARD-BRCT-CstF50 complex.

## Results and Discussion

### Oligomeric behavior of BARD1 ARD-BRCT

**Figure 1:**
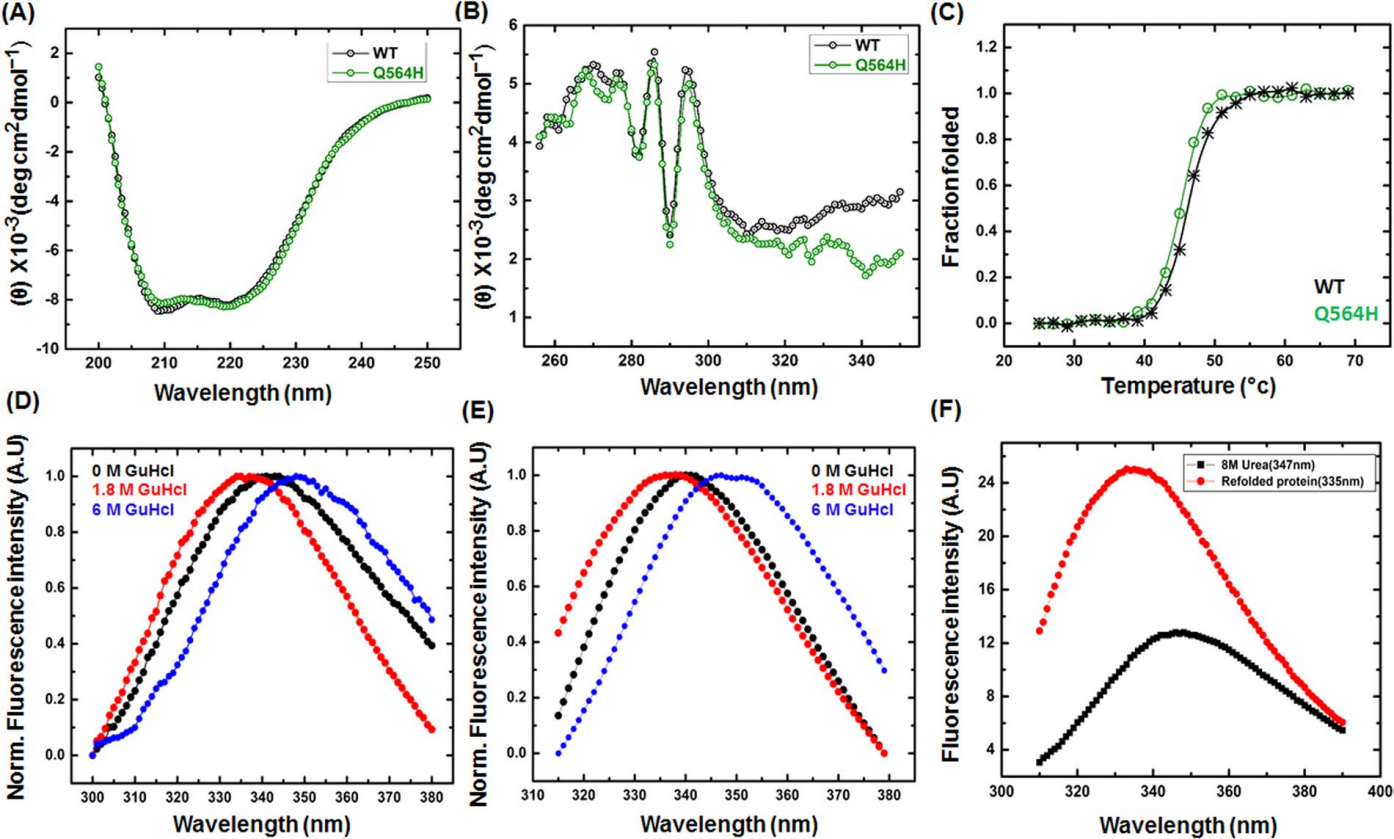
(**A** and **B**) Comparative Far -UV and Near -UV, CD spectroscopy profile of BARD1 ARD-BRCT *wild-type* and Gln 564 His mutant respectively, (**C**) Comparative thermal denaturation profile of *wild-type* and Gln 564 His mutant protein, (**D** and **E**) Comparative chemical denaturation profile of profile of *wild-type* and Gln 564 His mutant protein respectively and (**F**) Refolding profile of CstF50.

**Figure 2:**
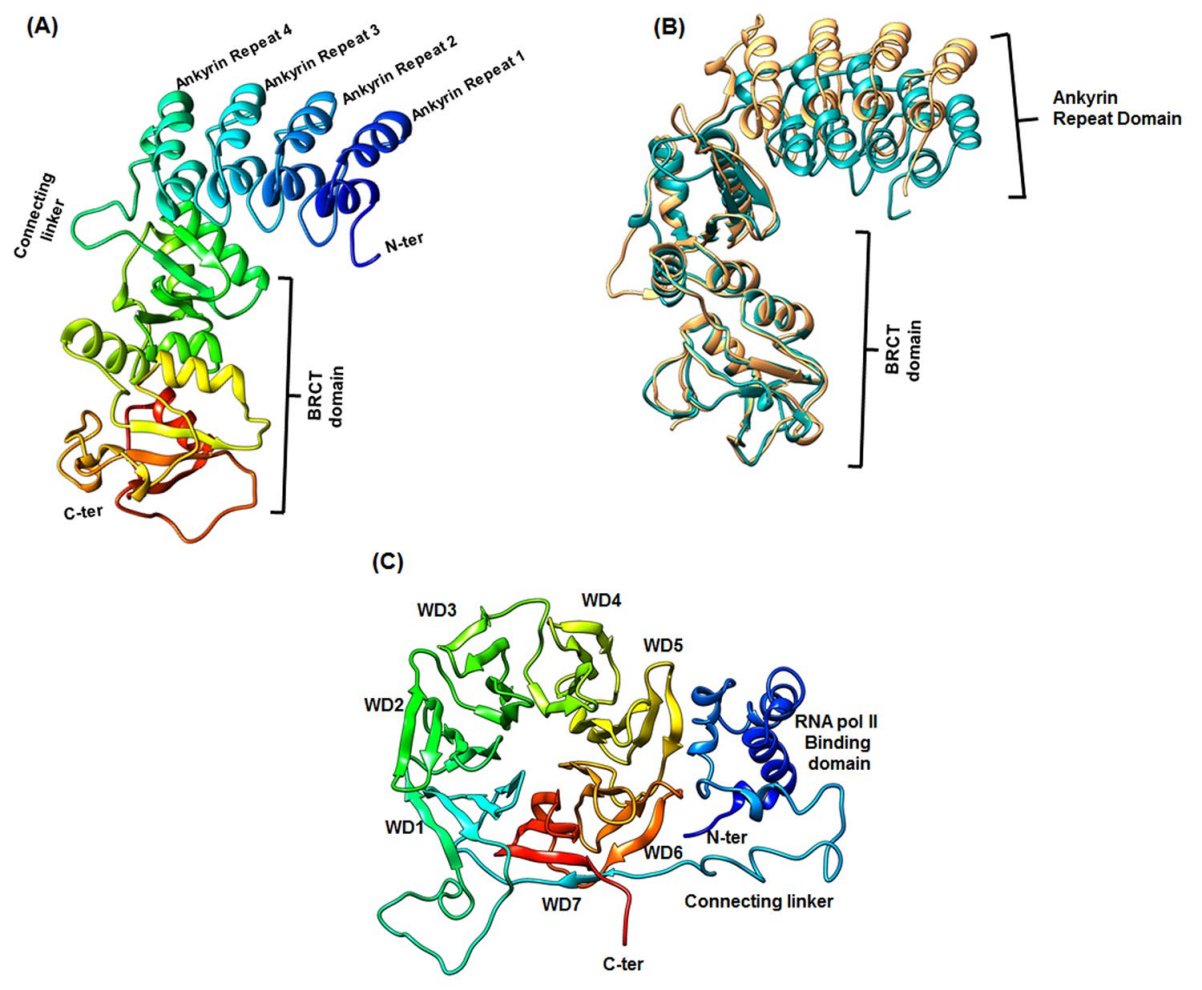
(**A**) Representative molecular model of BARD1 ARD-BRCT *wild-type*, (**B**) Comparative structural alignment profile of BARD1 ARD-BARD1BRCT wild-type (Navy Blue) and Gln 564 His Mutant (Yellow) and (**C**) Molecular Model structure of CstF50.

**Figure 3:**
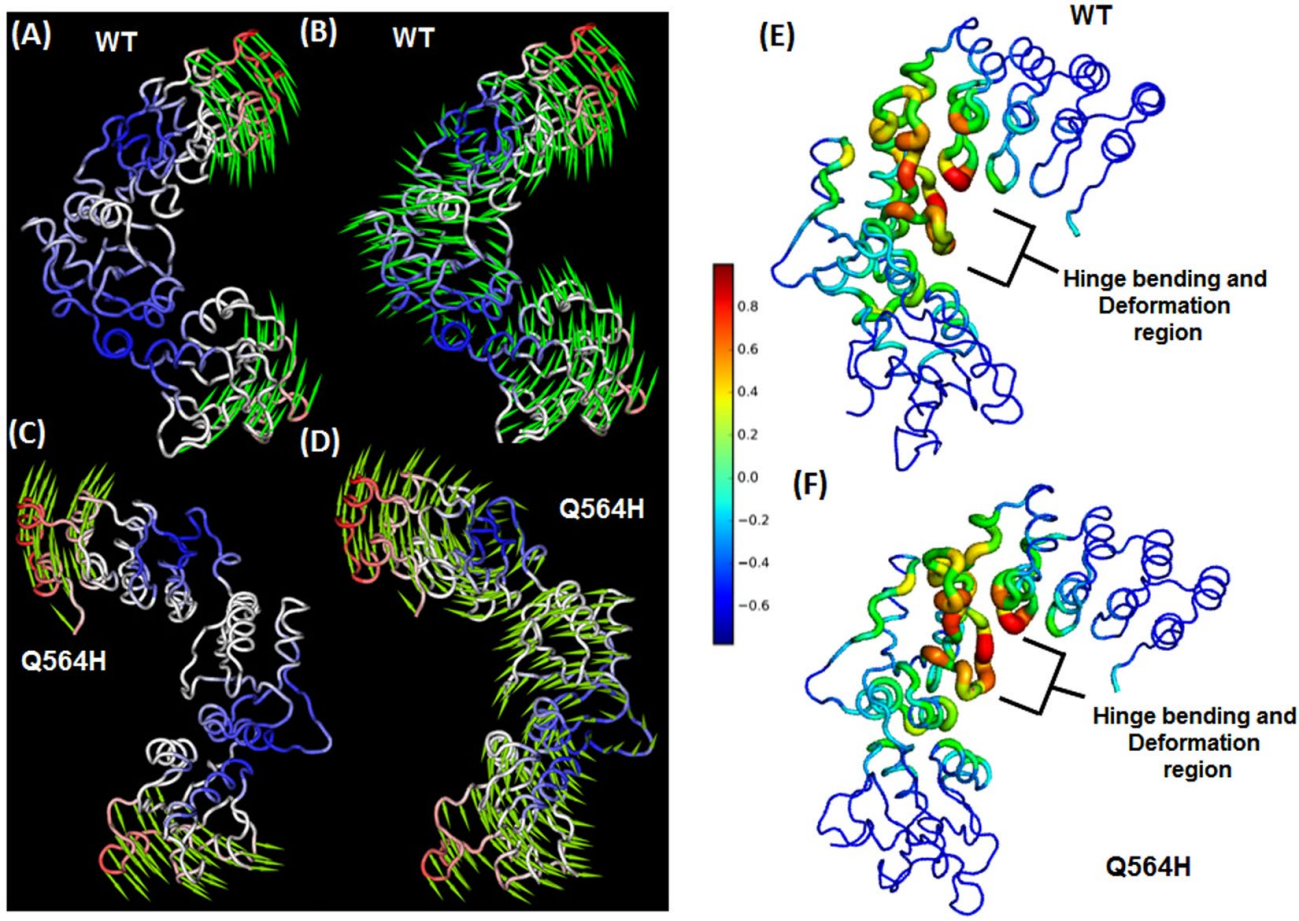
(**A** and **B**) Comparative Normal Mode Analysis and representation of mode 1 profile of BARD1 ARD-BRCT *wild-type*, (**C** and **D**) Gln 564 His mutant protein. (**E** and **F**) Comparative deformation analysis profile of BARD1 ARD-BRCT *wild-type* and Gln 564 His mutant protein.

To delineate the effect of mutation on the oligomeric behavior of BARD1 ARD-BRCT, the purified BARD1 ARD-BRCT *wild-type* and mutant proteins (Figure 1A,B Supplementary) were subjected to size exclusion chromatography and dynamic light scattering (DLS). The hydrodynamic radii of BARD1 ARD-BRCT *wild-type* and mutant protein from DLS experiment were 3.3 ± 0.45 nm and 3.38 ± 0.15 nm respectively which suggest that BARD1 Gln 564 His mutation does not affect the overall packing within the hydrophobic core of the protein (Figure 1E Supplementary). Both BARD1 ARD-BRCT *wild-type* and mutant proteins were eluted in the monomeric elution volume, suggesting their monomeric nature (Figure 2 Supplementary). Glutaraldehyde cross-linking assay for *wild-type* and mutant proteins do not show higher molecular weight conjugates on the 12% SDS-PAGE gel which further support the monomeric nature of proteins (Figure 3A and B Supplementary).

### ARD and BRCT domains of BARD1 are compact and folds independently

CD Spectroscopy (CD) was performed to explore the effect of mutation on the secondary structure of BARD1 ARD-BRCT. The CD spectra for *wild-type* and mutant protein shows that secondary structure of the protein is majorly stabilized by α-helices because of prominent ellipticity minima at λ = 208 nm and 222 nm (Fig. 1A). However, the CD spectra do not show change in the secondary structure of mutant protein which indicates that mutation has no effect on the secondary structure of the *wild-type* BARD1 ARD-BRCT protein. However, the absence of characteristic random coils and β-sheets are due to the dominant contribution of CD signal from the α-helix portion of the structure. Additionally, this masking of random coil and β-sheets can also be due to the disulfide chromophores present in ARD-BRCT region which strongly contributes to the CD signal in the range of λ = 215–235 nm^27, 28^. CD spectroscopy was further performed at near-UV range to investigate the effect of mutation on the tertiary structure of the protein. Near-UV spectra of *wild-type* and mutant proteins show peaks at λ_max_ = 295 nm and 285 nm respectively, which indicates the hydrophobic microenvironments of intrinsic fluorophores substantiating the compact 3D structure of the proteins^29^(Fig. 1B). Furthermore, limited proteolysis profile of *wild-type* and mutant protein indicates that ARD and BRCT domain of BARD1 are two compact and independently folding domains (Figure 3C and D Supplementary).

### Thermal stability of BARD1 ARD-BRCT

**Figure 4:**
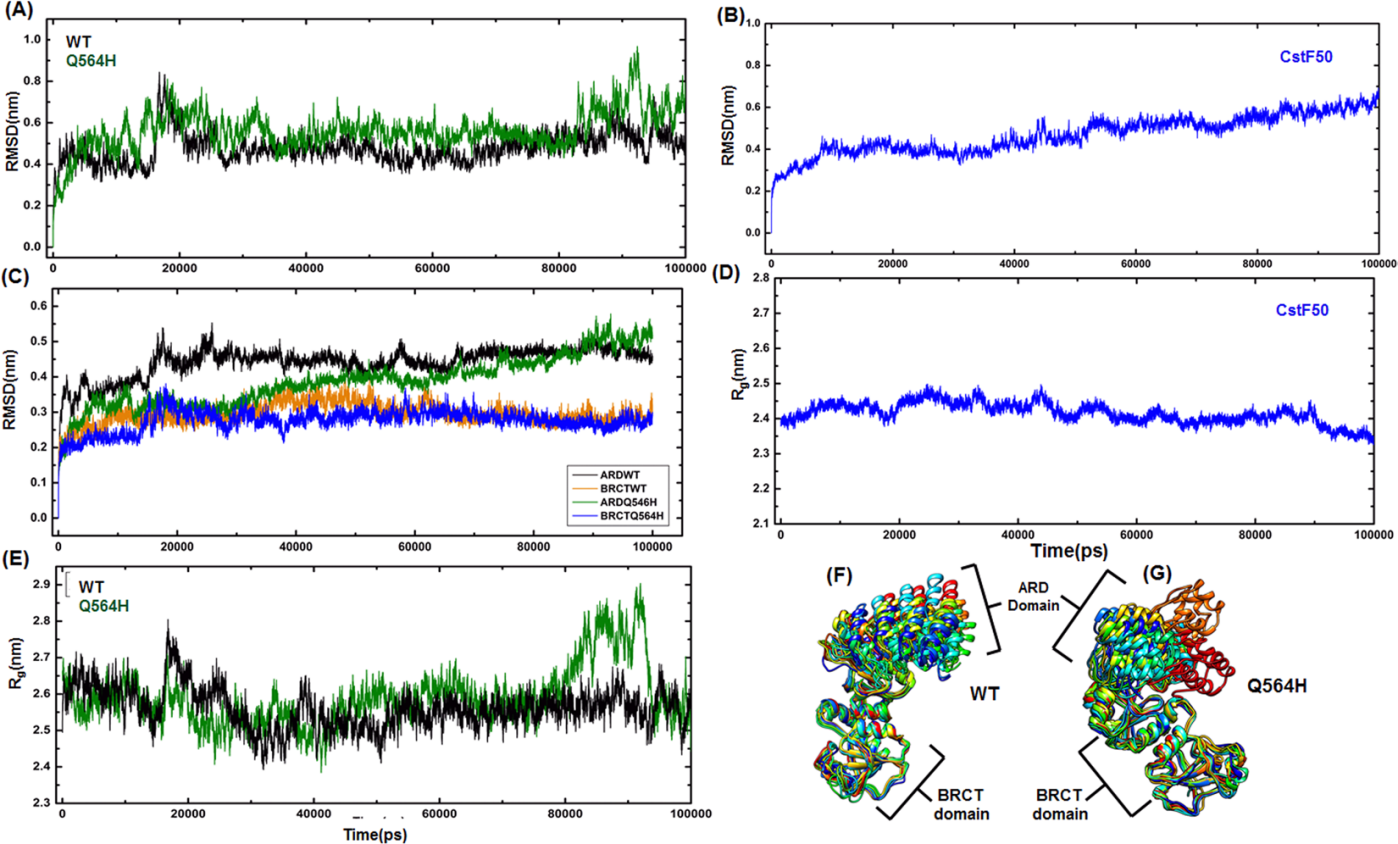
(**A** and **C**) Comparative RMSD and R_g_ profile of *wild-type* and Gln 564 His mutant protein respectively (**B** and **D**) RMSD and R_g_ profile of CstF50 respectively. (**E**) Individual RMSD profile of *wild-type* and Gln 564 His mutant protein ARD and BRCT domain respectively. (**F** and **G**) Comparative superimposition of 10 frames extracted from trajectory of *wild-type* and Gln 564 His mutant protein respectively.

Thermal denaturation was performed to delineate the effect of mutation on the thermal stability of the BARD1 ARD-BRCT *wild-type*. Thermal induced denaturation was monitored from 25 °C–65 °C using CD spectroscopy. The loss in secondary structure was observed at 65 °C for both the *wild-type* and mutant proteins (Figure 4A and B Supplementary). The unfolded fraction was calculated by using change in millidegree ellipticity at λ = 222 nm, which suggest that *wild-type* and mutant protein unfold via a two state pathway in a co-operative manner. The T_m_ calculated for *wild-type* and mutant were 45.6 ± 0.60 °C and 45.2 ± 0.42 °C respectively, which further suggest that mutation does not drastically affect the thermal stability of the BARD1 ARD-BRCT *wild-type* (Fig. 1C). Chemical denaturation induced by GuHcl was monitored using fluorescence spectroscopy. Fluorescence emission maximum at λ_max_ = 340 nm for *wild-type* and at λ_max_ = 341 nm for mutant indicate that proteins are in folded conformation. However, at 1.8 M GuHcl concentration significant blue-shifts in the emission maxima was observed for *wild-type* at λ_max_ = 335 nm and mutant at λ = 336 nm respectively. *Wild-type* and mutant completely unfold at 6 M GuHcl as indicated by the emission maximum at λ_max_ = 348 nm. These observations suggest that *wild-type* and mutant unfold via an intermediate molten globule formation (Fig. 1D and E), and (Figure 4C and D Supplementary).

Furthermore, to investigate the molecular interactions between CstF50 and BARD1, we have expressed His-tagged CstF50 in bacterial system that expressed mostly in inclusion bodies (Figure 1C and D Supplementary). Hence 6XHis tagged fused CstF50 protein was extracted using 8 M urea, and then further refolded in the buffer D (50 mM Tris, pH 6.2, 500 mM NaCl, 0.1% triton, 10% glycerol pH 7.2, 2 mM EDTA, 700 mM arginine, 100 mM KCl, 50 mM MgCl_2_, 100 mM CaCl_2_ and 5 mM DTT) containing Arginine. Refolding pattern of CstF50 protein was further confirmed by fluorescence spectroscopy. The fluorescence spectroscopy profile for denatured CstF50 in 8 M urea shows an emission maximum at λ_max_ = 347 nm, however, folded protein shows a blue-shifted emission maximum at λ_max_ = 337 nm, which indicates complete burial of tryptophans in the hydrophobic core of the protein (Fig. 1F). Furthermore, good quality CD spectra could not be achieved because arginine hydrochloride present in buffer D interfered with the measurement^30, 31^. An attempt to dialyze out arginine hydrochloride resulted in precipitation of CstF50 protein.

### CstF50 stabilizes the ARD domain of BARD1: *In-silico* approach

To understand the function associated to protein-protein interactions for BARD1-CstF50 complex, BARD1 ARD-BRCT region and CstF50 were modeled using Robetta server^32, 33^. This Model of BARD1 ARD-BRCT and CstF50 were further validated by MolProbity server^34^ (Table 1 and 2 Supplementary). The 3-D structure of BARD1 ARD-BRCT which comprises 4, N-terminal ankyrin repeats containing α-helices, a linker, two BRCT repeats containing 3-α helices and three β- strands (Fig. 2A). BARD1 ARD-BRCT Gln 564 His mutation was generated using SPDB viewer^35^. The BARD1 ARD-BRCT and Gln 564 His mutant structures were superimposed for direct comparison between *wild-type* and Mutant models (Fig. 2B). Modeled structure of CstF50 shows N-terminal helical region and C-terminal 7 WD-40 repeat comprised of beta sheets that fold into a propeller like structure (Fig. 2C).

Normal Mode Analysis (NMA) was performed on the BARD1 ARD-BRCT *wild-type* and Gln 564 His mutant structures to investigate the effect of mutation on protein dynamics. First few modes known as a low frequency modes describe the large domain motion within the protein^36^. Hence, to understand the structural dynamics between ARD and BRCT domain, we have selected the 1^st^ lowest frequency mode for BARD1 ARD-BRCT wild*-type* and Gln 564 His mutant protein structures (Fig. 3A–D). A large inter-domain motion was observed between the ARD and BRCT domain of BARD1. The inter-domain motion is often observed in multidomain proteins having hinge bending^37^. The residues at ARD and BRCT domain show high positive correlated motion at the terminal regions. Interestingly, the linker region residues show high anti-correlated motion for the ARD and BRCT domain residues in *wild-type* and mutant protein structures. The region of hinge bending and local flexibility were investigated using deformation analysis (Fig. 3E and F). The linker region was found to possess highest flexibility in both *wild-type* and mutant (thickness and color intensity) which shows that the linker between inter-domain region is responsible for large dynamics between ARD domain and BRCT domains.

### BARD1- ARD domain motion and CstF50 complex

**Figure 5:**
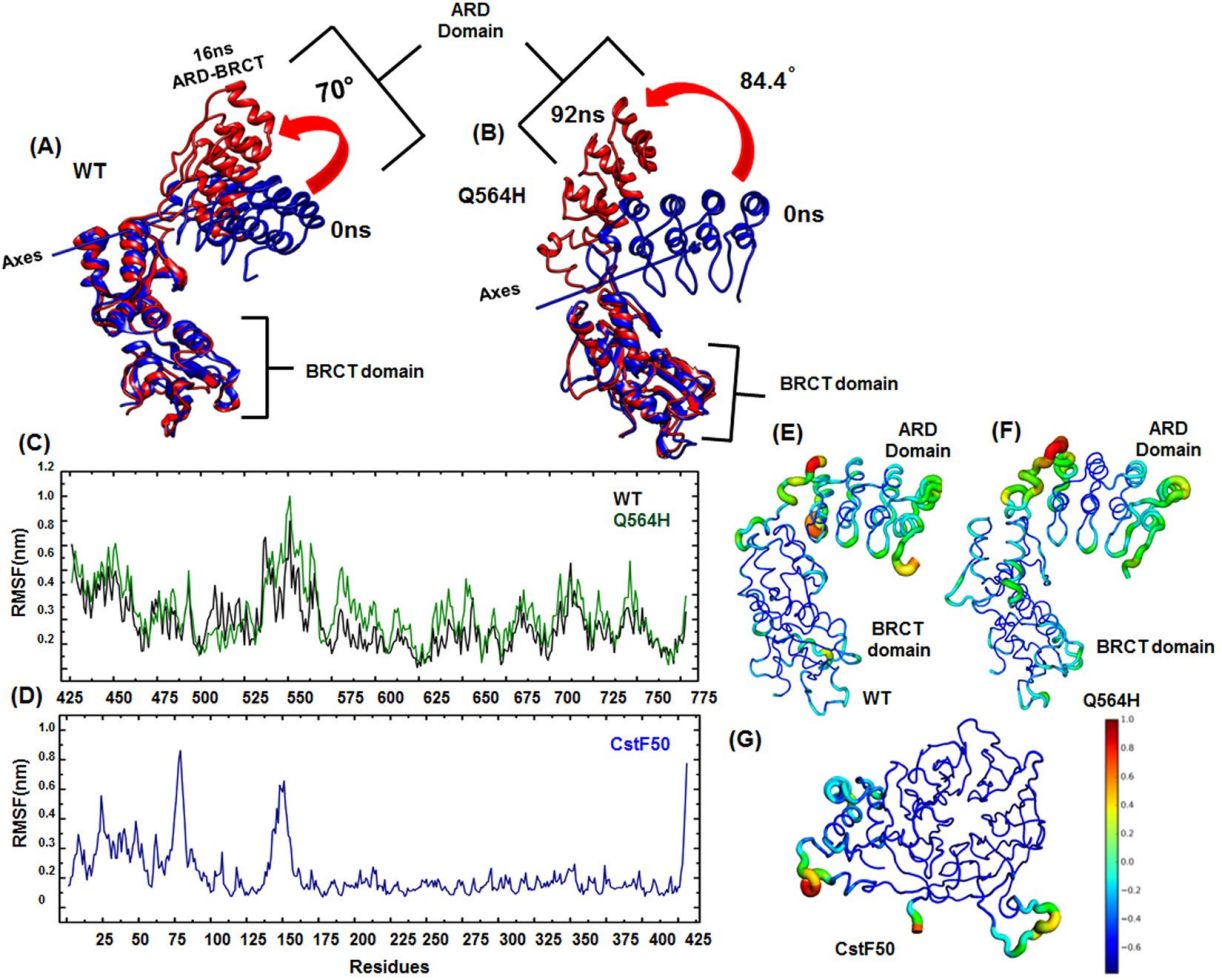
(**A** and **B**) Comparative superimposition of lowest RMSD and highest RMSD attained structure for *wild-type* and Gln 564 His mutant protein respectively (**C**) RMSF of *wild-type* and Gln 564 His mutant protein respectively. (**D**) RMSF profile of CstF50. (**E**,**F** and **G**) RMSF structure extracted from trajectory of *wild-type*, Gln 564 His mutant and CstF50 respectively.

To understand the function associated to protein-protein interactions of BARD1-CstF50 complex, and inter-domain motion between ARD and BRCT domains, the model structure of BARD1 ARD-BRCT, BARD1 Gln 564 His mutant, CstF50 and BARD1 ARD-BRCT-CstF50 complex were simulated for 100 ns under periodic boundary conditions. The RMSD (Root Mean Square Deviation) profile for BARD1 shows that mutant protein structure exhibits higher RMSD than the *wild-type* (Fig. 4A). During the first 5 ns of simulation, RMSD values of *wild-type* and mutant have increased up to a value of 5 Å. However, after 10 ns, RMSD of *wild-type* decreases to 3.5 Å and mutant increases up to 6.5 Å. At 16 ns, RMSD value for *wild-type* show sharp increase and reaches up to 8 Å. Hence, the mutant structure RMSD shows only a gradual rise to reach the level of *wild-type* RMSD (Fig. 4A). Beyond 16 ns, the decrease in the RMSD up to 4 Å for *wild-type* and mutant structure is indicative of structural stabilization. However, the RMSD of mutant protein structure remained high as compared to *wild-type*. After 35 ns of simulation, *wild-type* and mutant structures are stabilized for next 50 ns. Differences in RMSD were again observed at 98 ns and 96 ns of simulation for mutant and *wild-type* structures respectively. To investigate change in the RMSD, 0 ns input structures were superimposed onto the structure of 16 ns and 98 ns time frame for *wild-type* and mutant respectively. The two superimposed structures (Fig. 5A and B) show large ARD domain movement with respect to BRCT domain for *wild-type* and mutant protein structures. Superimposed structure of *wild-type* with input structure shows that ARD domain rotates about 70° relative to BRCT domain contributing to the RMSD value of 8.55 Å. However, the mutant shows that rotation of ARD domain by 84.4° leading to a RMSD of 9.27 Å with respect to the structure of 0 ns (Fig. 5A and B). The RMSD attained during the entire simulation by ARD domain of *wild-type* mutant shows that ARD domain is dynamic in nature comparative to the BRCT domain, which is stable during entire simulation (Fig. 4C). The RMSD change for ARD domain in mutant protein shows gradual increase but *wild-type* ARD domain is stable. Furthermore, 10 superimposed structures extracted for *wild-type* and mutant protein at different time points of trajectory show highly dynamic behavior of ARD domain, whereas the BRCT domain is stable in nature. The ARD domain in mutant shows conformational changes compared to *wild-type*, signifying that ARD domain in mutant protein is more dynamic than in *wild-type* (Fig. 4F and G). However, RMSD profile for CstF50 structure indicates that CstF50 is compact protein and does not show any changes in the model structure (Fig. 4B).

Furthermore, changes in the radius of gyration (R_gyr_) in a time dependent manner during the course of simulation determines the protein structure compactness^38^. During the early phase of simulation *wild-type* and mutant did not show any sharp changes in the R_gyr_, but steep rise in the R_gyr_ was observed at 16 ns in *wild-type* and at 98 ns in mutant structure of BARD1. In mutant structure, R_gyr_ change is significantly higher than that of *wild-type*, substantiating the inference that mutation has made the mutant protein dynamic than the *wild-type* structure (Fig. 4E).Change in R_gyr_ is largely due to change in the position of ARD domain relative to the BRCT domain of BARD1. However, R_gyr_ profile for CstF50 structure indicates that CstF50 has overall compact structure and does not show any changes in the conformation (Fig. 4D).

Comparative residual RMSF (Root Mean Square Fluctuation) values attained by *wild-type* and mutant suggest that the N-terminal ARD domain of BARD1 is highly flexible. The linker region (550–568)amino acids in BARD1 ARD-BRCT shows highest RMSF values in *wild-type* and Gln 564 His mutant structures (Fig. 5C). Hence, to explore the residual flexibility of BARD1 ARD-BRCT, RMSF structures showing maximum fluctuation from the trajectory were extracted (Fig. 5D and E). The RMSF structures for *wild-type* and mutant indicate that ARD domain is highly flexible, and high value of RMSF is observed for the residues in N-terminal, linker and loop regions of second BRCT repeat (Fig. 5C,E and F). However, in the CstF50 structure, RMSF indicated two flexible regions, first at the connecting loops between N-terminal and WD-40 domain, and second at the loop region between WD1 and WD2 repeat which also corroborates with the residual RMSF plotted for the CstF50 **(**Fig. 5D and G).

**Figure 6:**
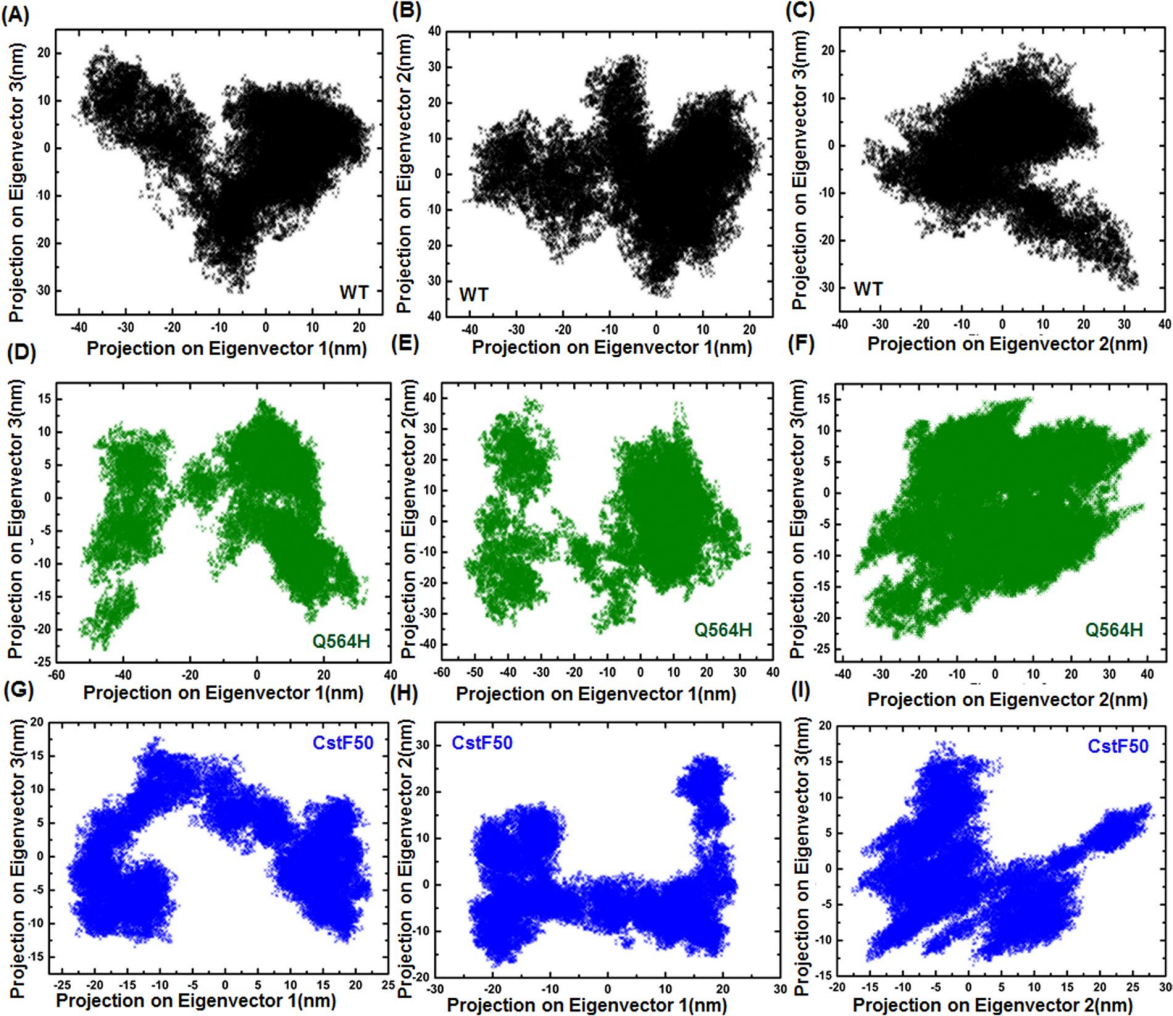
(**A**,**B** and **C**) Comparative Eigenvector projection profile of BARD1 ARD-BRCT *wild-type* (**D**,**E** and **F**) Gln 564 His mutant protein respectively (**G**,**H** and **I**) CstF50 respectively.

Furthermore, domain movement of the protein structures have been studied in relation to possible involvement with protein function^39^. To probe the collective motion during the simulation, the principal component analysis (PCA) was performed over the BARD1-ARD-BRCT, Gln 564 His mutant, CstF50 and BARD1-ARD-BRCT-CstF50 complex trajectories. The Eigenvalues and eigenvectors were calculated after the diagonalization of the covariance matrix. The area of trace of covariance matrix calculated for BARD1-ARD-BRCT, Gln 564 His mutant and CstF50 were 487.990 nm^2^, 714.963 nm^2^ and 540.810 nm^2^ respectively, which indicates that mutant shows more dynamic tertiary structural conformations than the *wild-type* protein. Hence, the trajectories for domain motion of BARD1 ARD-BRCT *wild-type*, Gln 564 His mutant and CstF50 were projected on the first three eigenvectors. Projection on eigenvector 2 and 1 for *wild-type* and mutant protein indicates that mutant protein is more dynamic in nature than the *wild-type*. Trajectory Projection on eigenvectors combination 3 and 1, 2 and 1 and 3 and 2 for *wild-type* (Fig. 6A,B and C) and mutant (Figure 6D,E and F) indicates that both the structures can attain three major tertiary structure conformations whereas mutant structure shows higher periodic jump. Further, projection of eigenvectors 3 and 1, 2 and 1 for CstF50 shows that two major groups of tertiary structural conformation can be attained by the CstF50. The large transitions and periodic fluctuation between these conformers throughout the trajectory have been observed (Fig. 6G,H and I).

### Comparative residual displacement along the Eigenvectors

To investigate the residual fluctuation, first two eigenvector extracted from trajectories of BARD1 ARD-BRCT *wild-type*, Gln 564 His mutant and CstF50 were projected on the residues. The plot of projection on eigenvector 1 and 2 of residues for *wild-type* (Figure 5A,B Supplementary) and mutant (Figure 5C,D Supplementary) demonstrates concerted residual fluctuation, which shows high flexibility in ARD domain, linker region and loop regions of BARD1 BRCT domain. Furthermore, eigenvector 1 and 2 were also projected on residue for CstF50 (Figure 5E,F Supplementary). The projection of eigenvector 1 and 2 show different profile of residual fluctuation and do not shows any similarity. The projection of eigenvector 1 on residue shows flexible loop regions connecting N-ter, WD-40 domain. On the other hand projection of eigenvector 2 on residues shows stable WD-40 domain but fluctuations in the N-terminal domain that may be due to the connecting loop regions and untethered N-terminal (Figure 5E,F Supplementary).

### Positive correlation motion of BARD1 ARD domain

**Figure 7:**
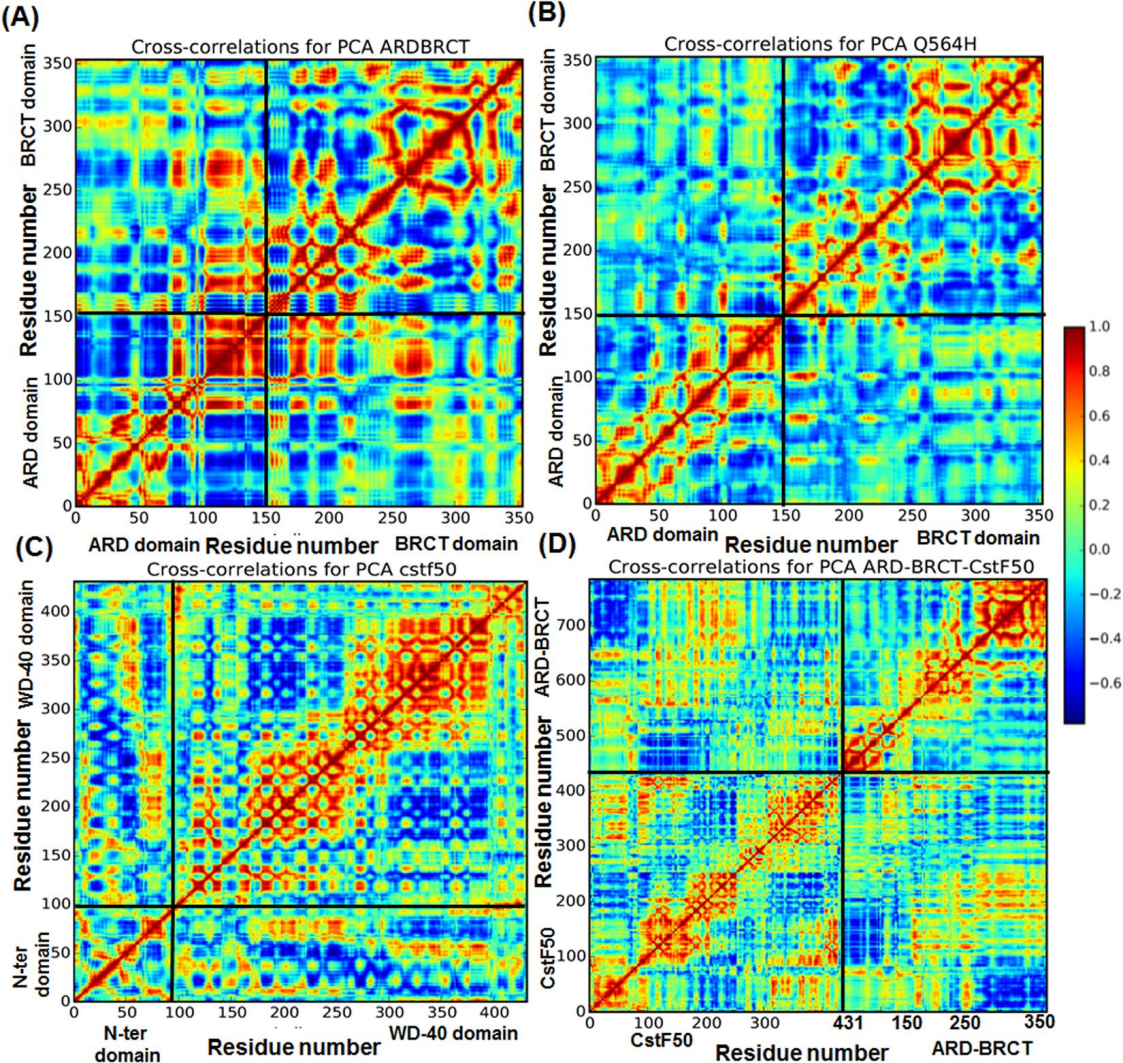
Comparative residual cross-correlation for PCA of (**A**) BARD1 ARD-BRCT *wild-type*, (**B**) Gln 564 His mutant, **(C)** CstF50 respectively and (**D**) BARD1 ARD-BRCT *wild-type* -CstF50 complex respectively. Red and yellow regions show highly positively correlated regions while dark blue represent anti-correlated movement of residues.

Cross correlation for PCA of BARD1 *wild-type*, Gln 564 His mutant protein, CstF50 and complex was plotted to investigate the correlated motion within the proteins. Cross-correlation of PCA indicates high positive correlation within the ARD domain. The residues within the ARD domain show strong negative correlation with the linker region which is in consistent with the NMA results (Fig. 7A,B). Comparatively, BARD1 *wild-type* shows higher positive correlation motion within the ARD domain and linker region residues as compared to the mutant protein. Furthermore, mutation has also affected the positive correlation within the BRCT repeat as *wild-type* shows higher positive correlation within the N-terminal BRCT and C-terminal repeat residues. CstF50 cross-correlation for PCA indicates that the N-terminal residues show little positive correlation within themselves as compared to the WD-40 repeat domain, which shows very high positive correlated motion within their domain (Fig. 7C). However, BARD1 ARD-BRCT-CstF50 complex cross-correlation for PCA indicates increased positive correlated motion within the N-terminal residues and WD-40 repeat for CstF50 in complex as compared to native condition. Furthermore, higher positive correlation is also observed in the ARD domain as well as BRCT domain in complex compared to the BARD1 ARD-BRCT alone (Fig. 7D).

### Protein-Protein Interactions between BARD1 ARD-BRCT and CstF50

The minimum free energy model structures of BARD1 ARD-BRCT, Gln 564 His mutant and CstF50 were selected for protein-protein docking studies (Figure 6A,B and C Supplementary). It is well established that 7^th^ WD 40 (395–431) repeat domain of CstF50 is required to establish BARD1 complex^26^. The Gln at 564 to His mutation reduces the binding affinity of CstF50 to BARD1^17^. Therefore, we considered BARD1 Gln 564 as an important residue for interaction study with CstF50. In-silico docking was performed to understand the residual interactions between BARD1 ARD-BRCT, Gln 564 His mutant with CstF50. In the intermolecular interface of BARD1-CstF50 complex, glutamine 564 of BARD1 ARD-BRCT forms hydrogen bond with Asn 409 and Tyr 425 of CstF50, and also participates in non-hydrogen bonding interactions with Tyr 425, Asn 409, Pro 410, and Thr 408 of CstF50. The other BARD1 residues Thr 562 and Gly 563 also form hydrogen bonds with Thr 430, Tyr 425 and Ser 428 of CstF50 respectively (Figure 7A and B Supplementary). However, in the BARD1 Gln 564 His mutant-CstF50 complex, BARD1 His 564 lost hydrogen bonding interactions but forms non-covalent interactions with Gly 394 and Leu 395 of the CstF50 (Figure 7C and D Supplementary). The BARD1 Thr 562 was found to be involved in the non-covalent interactions with CstF50 Glu 100. Loss of weak intermolecular interactions in case of mutant rationalizes the loss of binding affinity between mutant BARD1 and CstF50 (Figure 7D Supplementary).

### Binding Interface of BARD1 ARD-BRCT and CstF50 complex

**Figure 8:**
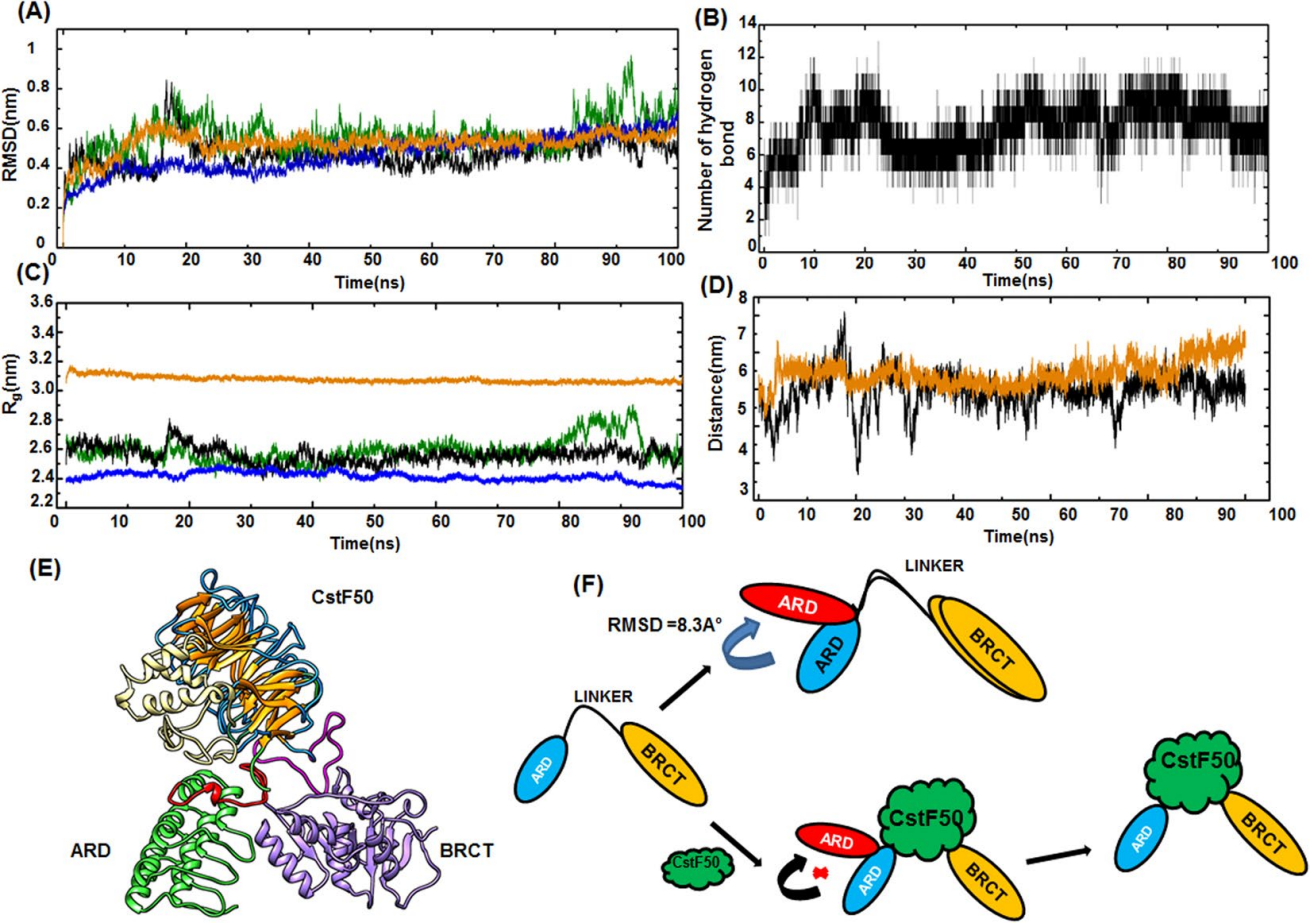
(**A** and **C**) RMSD and R_g_ profile of BARD1 ARD-BRCT *wild-type*, Gln 564 His mutant, CstF50 and *wild-type-*CstF50 complex respectively (**B**) H bonding profile of BARD1 ARD-BRCT *wild-type-*CstF50 complex (**D**) distance fluctuation profile of ARD domain in BARD1 ARD-BRCT *wild-type* and complex (**E**) Structure of *wild-type-*CstF50 complex and (**F**) Model for mechanism of *wild-type-*CstF50 complex stabilization.

To determine the stability of BARD1 ARD-BRCT-CstF50 complex molecule we have simulated the structure for 100 ns to analyze the comparative RMSD, RMSF, R_gyr_ and hydrogen bonding (Fig. 8). Comparative RMSD and R_gyr_ indicate that binding of CstF50 to the BARD1 ARD-BRCT restricts the ARD domain fluctuation relative to the BRCT domain (Fig. 8A and C). Hydrogen bonding analysis between the linker region and 7^th^ WD-40 domain was performed to analyze the domain stability of complex (Fig. 8B). It was found that BARD1 ARD-BRCT *wild-type* linker and 7^th^ WD-40 repeat form stable hydrogen bonds throughout the simulation, thus enabling formation of a stable complex (Fig. 8D). Further, binding energy and other energy component calculated for BARD1-CstF50 complex for entire simulation indicated that interaction between BARD1 and CstF50 is stable (Table 4 Supplementary). Interestingly, interactions between CstF50 and the BARD1 linker rigidifies the linker flexibility and thereby controls the architectural placement of the BARD1 C-terminal domain relative to the N-terminal ARD domain (Fig. 8E). This phenomenon thereby reduce the relative inter domain flexibility of the ankyrin repeats and BRCT domains. The short length of the linker and germ-line cancer-predisposing mutations suggest that the relative positioning of ARD and BRCT domains within BARD1 is critical in the binding to proteins and functionality of BARD1 (Fig. 8E and F).

## Conclusion

Multidisciplinary approaches were applied to characterize the domain motion and binding affinity between BARD1 ARD-BRCT, Gln 564 His mutant and CstF50 proteins. It has been observed that the BARD1 ARD-BRCT Gln 564 His mutant is not affecting the monomeric property, secondary structure, hydrodynamic radii and thermal stability of the *wild-type* protein. The limited proteolysis of *wild-type* and mutant confirms that ARD and BRCT domain are stable and independent folded domains of BARD1 connected by a short stretch of a linker region. These results from limited proteolysis are consistent with earlier NMR studies which conclude that in the absence of any interacting protein, the ARD and tandem BRCT region behave as an independent domain in solution^40, 41^. The thermal unfolding study indicates that BARD1 ARD-BRCT *wild-type* and mutant unfold via a two state pathway. However, chemical unfolding of *wild-type* and mutant suggest that both proteins unfold via a molten globule intermediate.

The results from NMA and MD simulation substantiates well with the earlier reported short angle X-ray scattering (SAXS) findings. In solution ARD and BRCT domains sample a wide range of relative orientations with respect to one another^42^. The Small Angle X-ray Scattering (SAXS) for BARD1 ARD-BRCT domain shows two additional features in the p(r) function: a shoulder at r ∼40 Å and tailing-off of the at r > 90 Å^42^. SAXS for ARD-BARD1BRCT suggests that Dmax of the domain is around 90 Å^42^. The RMSD values calculated for *wild-type* and mutant are very close (87 Å) to the experimental values (r > 90 Å)^42^. The distance calculated for *wild-type* N-terminal ARD domain and C-terminal BRCT domain shows a maximum change of 40 Å, which shows dynamic behavior of the protein due to the flexible linker region. The NMA and deformation analysis indicate that the linker connecting ARD and BRCT domains makes the *wild-type* and mutant protein flexible. MD simulation studies show that ARD domain is highly dynamic than the BRCT domain due to flexible linker which acts as the connecting bridge between the ARD and BRCT domain of BARD1. Flexibility in linker segments of the BARD1 ARD-BRCT backbone allow corresponding domain motions to occur with minor structural perturbations. The RMSD, R_gyr_ and RMSF profiles show that BARD1 Gln 564 His mutant protein structure is more flexible than the *wild-type*. The relative orientation of ARD domain is significantly affected due to the Gln 564 His mutation. In BARD1 ARD-BRCT domain, flexible linker has different orientation that allows free twist and rotation of the ARD domain through space which can facilitates the recruitment of CstF50. The mutation Gln 564 His is present in the disordered flexible linker region of BARD1 ARD-BARD1BRCT domain. Lack of specific secondary structure provides the accessibility of different orientations to the linker region. The increase in the relative flexibility may be due to twist in the linker region by histidine. The linker region acts as a binding region for two large proteins CstF50 and p53. The difference in hydrogen bond donor group in glutamine and histidine may be the other reason in the loss of BARD1-CstF50 interactions. The results from *wild-type*, mutant and CstF50 docking study indicates that glutamine 564 to histidine mutation shows loss in hydrogen bonding interactions between *wild-type* and CstF50. To our conclusion, BARD1 Gln at 564 position plays a pivotal role in the BARD1 ARD-BRCT-CstF50 complex formation. The study further shows CstF50 binding provides rigidity to the ARD domain of BARD1. Therefore, flexibility in the linker region is immensely required to adopt such unique orientation to accommodate CstF50 protein.

## Material and Methods

All the chemicals used in this study were purchased form Sigma-Aldrich, unless otherwise specified. The buffers were prepared in double distilled, 0.44 µM filtered Milli-Q water (Millipore, USA). Filtered 0.44 µM (Millipore, USA) protein and buffer solutions were degassed prior to use.

### Protein expression and purification

BARD1 ARD-BRCT (425–777) cloned into a modified pET-6H vector (generous gift by Dr. Richard Baer, Institute for Cancer Genetics, Columbia University, USA). BARD1 ARD-BRCT *wild-type* domain was further PCR amplified and cloned into pGEX-KT vector (Amersham) in such a way that TEV protease site was incorporated between the GST and BARD1 ARD-BRCT. Site directed mutagenesis was performed to engineer the Gln 564 His mutation in BARD1 ARD-BRCT, and further sequenced for confirmation. BARD1 ARD-BRCT *wild-type* and Gln 564 His mutant were expressed in *Escherichia coli* BL21 (DE3). The GST-BARD1 ARD-BRCT *wild-type* and Gln 564 His mutant fusion protein were purified using affinity chromatography (GST-sepharose 4B column) in 50 mM Borate buffer, pH 9.0, 300 mM NaCl(Buffer A). The affinity purified proteins were treated with TEV to remove the fusion tag. Molecular exclusion chromatography was further performed to achieve high purity and homogenous monomeric population of BARD1 ARD-BRCT *wild-type* and Gln 564 His mutant proteins. Superdex-75 column was used for the purification of the BARD1 ARD-BRCT *wild-type* protein. The Mutant protein was purified using the superdex-200 analytical column.

Human-CstF50 cDNA was bought from DNASU cDNA repository (Arizona state university) was PCR amplified and cloned in to pET-28a vector (Invitrogen). CstF50 cloned in pET28a vector was expressed in *Escherichia coli BL21* (*DE3*) cells grown at 37 °C until absorbance at λ = 600 nm reached a value in between 0.6–0.8, followed by induction with 0.1 mM IPTG at 18 °C for 18 hrs. Most of the CstF50 was found as insoluble aggregates at 18 °C when induced with 0.1 mM (IPTG). The insoluble aggregate of CstF50 was washed with buffer composition 50 mM, 500 mM NaCl, 5% glycerol, 1% sarkosyl, 1% SDS, pH 8 (Buffer B) by resuspending the pellet and again centrifuging at 18000 r.p.m. After washing, pellet was resuspended in 50 mM, 500 mM NaCl, 8 M urea, 5% glycerol (Buffer C), pH 8 for 5 hours to solubilize completely. Then 8M-urea-solubilized-CstF50 was refolded using buffer 50 mM Tris, 500 mM NaCl, 0.1% triton, 10% glycerol pH 7.2, 2 mM EDTA, 700 mM arginine, 100 mM KCl, 50 mM MgCl_2_, 100 mM CaCl_2_, and 5 mM DTT, pH 6.2 (Buffer D). In the refolding process we have added 10% glycerol and 0.5 M arginine hydrochloride to improve the refolding process^43, 44^. To overcome the aggregate formation, high concentration of arginine was used to suppress aggregation of folding intermediates.

### Circular - Dichroism spectroscopy

Circular dichroism spectra for BARD1 ARD-BRCT *wild-type* and Gln 564 His mutant were collected using 0.1 cm path length sealed quartz cuvette on a JASCO J-715 spectropolarimeter (Jasco, Easton, MD), equipped with a JASCO PTC 348 WI temperature controller. BARD1 ARD-BRCT and Gln 564 His mutant at a concentration of 15 μΜ were scanned in the far- UV (λ = 180–260 nm) and near- UV range (λ = 350–260 nm). Seven spectra were collected with 20 nm scan speed at a resolution of 1 nm and, response time of 1 s was averaged for each experiment. Blank spectra using buffer E (50 mM Borate buffer, pH 9.0, 150 mM NaCl) have been taken under the similar conditions and subtracted from the raw data. The spectra was recorded at the 25 °C. The results have been expressed terms of molar ellipticity [θ] (deg cm^2^ dmol^−1^).

### Limited proteolysis

BARD1 ARD-BRCT *wild-type* and mutant at a concentration of 2 mg/ml were incubated with the 10 Þg/µl trypsin in different time intervals of 0, 5, 10, 30, 60 and 120 mins at 37 °C. After incubation, reaction was terminated by adding 1 mM PMSF (sigma Aldrich). Samples collected at different time intervals were analyzed over SDS-PAGE gel. BARD1 ARD-BRCT *wild-type* and Gln 564 His mutant protein untreated with trypsin were taken as control.

### Glutaraldehyde crosslinking

BARD1 ARD-BRCT *wild-type* and Gln 564 His mutant at a concentration of 0.5 mg/ml in buffer A (50 mM Borate buffer, pH 9.0, 300 mM NaCl) was incubated with freshly prepared solution of glutaraldehyde (final concentration 0.1%) for 0, 1, 2, 3, 5, 10, 15 and 30 minutes at 37 °C. Crosslinking reaction was terminated by adding 5 μl of 1 M Tris-HCl, pH 8.0, and the samples were analyzed on 12% SDS-PAGE gel.

### Dynamic light scattering

Molecular size measurement and oligomeric behavior of BARD1 ARD-BRCT *wild-type* was performed using Malvern Zetasizer (Malvern). BARD1 ARD-BRCT *wild-type*, Gln 564 His mutant protein, and buffer were filtered (0.22 µm), degassed thoroughly prior to every measurement. BARD1 ARD-BRCT *wild-type* protein and mutant protein at a concentration of 1 mg/ml were scanned at an interval of 5 minutes for 15 minutes. The scanning were performed at the 25 °C. The DLS experiments were repeated in three independent sets.

### Thermal and chemical denaturation

Thermal denaturation was monitored by circular dichroism spectroscopy. BARD1 ARD-BRCT *wild-type* and Gln 564 His mutant were allowed to unfold over the temperature ranges from 20 °C to 70 °C. Further fraction unfolded was calculated at each temperature by using millidegree ellipticity at λ_222_ and data was fitted in to a two state unfolding pathway. BARD1 ARD-BRCT *wild-type* and mutant Gln 564 His were allowed to unfold by incubating 2 μM protein in different concentrations of GuHcl at 10 °C for 18 hrs. Chemical denaturation of BARD1 ARD-BRCT *wild-type* and Gln 564 His mutant were performed at 10 °C and the fluorescence from frequently used fluorophores was monitored using fluorescence spectrophotometer (Horiba, USA) at excitation wavelength of λ = 295 nm. BARD1 ARD-BRCT *wild-type* and Gln 564 His mutant at a concentration of 2 μM was mixed with the GuHcl. The concentration of GuHcl varied from 0 M to 6 M, while the protein concentration was fixed at 2 μM. Fluorescence emission spectra were recorded over the range of wavelength λ = 310–400 nm in order of increasing GuHcl concentration, and blank subtraction was done to increase signal to noise ratio.

### Molecular dynamics simulation

The validated Model structures of BARD1 ARD-BRCT *wild-type*, mutant Gln 564 His, CstF50 and BARD1-CstF50 complex were subjected to MD simulations using GROMACS 4.5.5^45–47^ software. Protein were solvated with explicit solvent Single Point Charge water, in a cubic box which left 2 Å space nearby the solute. The counter ions in the form of Na^+^ and Cl^−^ ions were added to make the system electrically neutral. Further energy minimization by the steepest descents method was used, and system was coupled to an external bath using Berendsen’s method. The reference temperature for all simulation was fixed at 300 K. LINC method was used to constrain all bonds^48, 49^. OPLS-AA force field in GROMACS was used and grid type neighbor searching was done, and long range electrostatics was controlled using PME^50, 51^. During simulations, the respective energy minimized structures were subjected to a position restrained MD Simulation for 100 ns with a time step of 2 fs. The trajectories were saved at every 2 ps to form respective trajectories. GROMACS built in tools were used to calculate RMSD (Root Mean Square Deviation), RMSF (Root Mean Square Fluctuations) and R_gyr_ (radius of gyration).

### Normal mode, Deformation and fluctuation analysis

Normal mode, deformation and fluctuation analysis were performed to study the domain motion in BARD1 ARD-BRCT *wild-type* and mutant protein using R 3.2 bio3D package^52^. First five modes were calculated by building the hessian matrix, and further extracted to explain the relative fluctuation in ARD domain of *wild-type* and mutant protein.

### Principal component analysis (PCA)

PCA reduces the dimensionality of the MD trajectory data and provides a concise way to visualize, analyze and compare large-scale concerted motions observed over the course of the simulation. PCA was performed using GROMACS in-built tool g_covar, g_anaeig and covariance matrix was built after removing net translational or rotational motion of the system by fitting the coordinate data to a reference structure. Further, diagonalization of the symmetric 3 *N* × 3 *N* covariance matrix was performed via eigenvector decomposition method and an orthogonal set of eigenvectors which are also called as functional “modes” were calculated with eigenvalues.

### Molecular docking

BARD1 ARD-BRCT *wild-type*, Gln 564 His and CstF50 docking was performed using HADDOCK server 2.0 which uses data-driven method of docking, with support from an extensive variety of experimental data obtained from diverse biophysical and biochemical experimental method^53^.

### Binding energy calculation

In common term binding free of complex in solvent can be calculated by **∆G**_**Binding**_ = **G**_**complex**_ **−**(**G**_**protein**_ + **G**_**Ligand**_). Free energy and other energy parameters of the complex were calculated by using molecular mechanics Poisson-Boltzmann surface area (MM-PBSA) method in GROMACS 4.5.5 package. *g_mmpbsa* tool was used to integrate high-throughput molecular dynamics simulation with the estimation of free energy of interaction^54^.

## Electronic supplementary material

Supplementary Dataset 1
