# Supplementary material for: Structural basis to stabilize the domain motion of BARD1-ARD BRCT by CstF50
Source: Sci Rep. 2017 Jun 20;7:3849. doi: 10.1038/s41598-017-03816-4 (PMC5478621; doi:10.1038/s41598-017-03816-4)

**Structural basis to stabilize the domain motion of BARD1-ARD BRCT by CstF50**

Rajan Kumar Choudhary^a,b^, Mohd Quadir Siddiqui^a,b^, Pankaj S Thapa^a,b^, Nikhil Gadewal^a^ , Senthil Kumar Nachimuthu^c^, Ashok K Varma^a,b,*^

^a^Advanced Centre for Treatment, Research and Education in Cancer, Kharghar, Navi Mumbai, Maharashtra 410 210, INDIA.

^b^Homi Bhabha National Institute, Training School Complex, Anushaktinagar, Mumbai - 400 094

^C^ Department of Biotechnology, Mizoram University (A Central University) Aizawl – 796 004, Mizoram, INDIA

**Keywords:** Protein-Protein Interactions, BARD1-ARD BRCT, CstF50

**Short-title:** Domain motion of BARD1-ARD BRCT and CstF50 complex

Address for Correspondence

٭ASHOK K VARMA Ph.D

Principal Investigator & Scientific Officer 'F'

Tata Memorial Centre,

Advanced Centre for Treatment, Research and Education in Cancer,

Kharghar, Navi-Mumbai, Maharastra Strate 410 210, INDIA.

Email: [avarma@actrec.gov.in](mailto:avarma@actrec.gov.in)

**Supplementary Table 1:-** Predicted secondary structure in percentages for BARD1 ARD-BRCT wild-type and mutant protein using K2D server

| **BARD1 ARD-BRCT wild-type on the basis of Mean Residual Ellipticity (MRE)** | **Mutant Gln 564 His on the basis of Mean Residual Ellipticity (MRE)** |
| --- | --- |
| α helix: 73.57% | α helix: 73.64% |
| β strand: 1.85% | β strand: 1.85% |

**Supplementary Table 2**: -Stereochemistry details of BARD1 ARD1-BARD1BRCT Model

| Poor rotamers | 2 | 0.64% | Goal: <1% |
| --- | --- | --- | --- |
| Ramachandran outliers | 0 | 0.00% | Goal: <0.05% |
| Ramachandran favored | 344 | 98.01% | Goal: >98% |
| Cβ deviations >0.25Å | 1 | 0.30% | Goal: 0 |
| Bad backbone bonds: | 0 / 1411 | 0.00% | Goal: 0% |
| Bad backbone angles: | 1 / 1762 | 0.06% | Goal: <0.1% |

**Supplementary Table 3**: -Stereochemistry details of CstF50 Model

| Poor rotamers | 3 | 0.79% | Goal: <1% |
| --- | --- | --- | --- |
| Ramachandran outliers | 0 | 0.00% | Goal: <0.05% |
| Ramachandran favored | 400 | 93.24% | Goal: >98% |
| Cβ deviations >0.25Å | 0 | 0% | Goal: 0 |
| Bad backbone bonds: | 0 / 1723 | 0.00% | Goal: 0% |
| Bad backbone angles: | 2 / 2152 | 0.09% | Goal: <0.1% |

**Supplementary Table 4**:- Binding free energy term between BARD1 ARD-BRCT and CstF50 calculated using MM-PBSA method

| Energy component | Calculated values |
| --- | --- |
| Van der Waal energy | -679.810 +/- 94.195 kJ/mol |
| Electrostatic energy | -1157.259 +/- 159.008 kJ/mol |
| Polar solvation energy | 1702.183 +/- 183.057 kJ/mol |
| SASA energy | -95.981 +/- 8.524 kJ/mol |
| Binding energy | -230.866 +/- 126.668 kJ/mol |
| ∆G_bind_= ∆ Van der Waal energy+∆ Electrostatic energy+∆ Polar solvation energy+∆ SASA energy-T∆S | |

**Supplementary Figure 1: -** Expression, Purification profile of (A) BARD1 ARD-BRCT *wild-type*, (**B**) Gln 564 His mutant and (**C, D**) CstF50. 1(**E**) shows comparative hydrodynamic radii calculation of BARD1 ARD-BARD1BRCT *wild-type* and Gln 564 His mutant. **Figure A** (1- Marker, 2- Uninduced whole cells, 3 – Induced whole cells, 4-Induced Soluble fraction, 5- Beads After Binding, 6- Cleaved protein) **Figure B**. (Lane 1- Marker Lane 2- Induced whole cells, Lane 3 – Induced Soluble fraction, Lane 4- Beads After Binding, Lane 5- Cleaved protein). **Key**:- **Figure** **C** (1- Marker, 2- Uninduced whole cells, 3– Induced whole cells, 4-Induced Soluble fraction, 5- Beads after binding, 6- Pellet). **Figure D** (1-Marker, 2-10 Purified CstF50 protein)


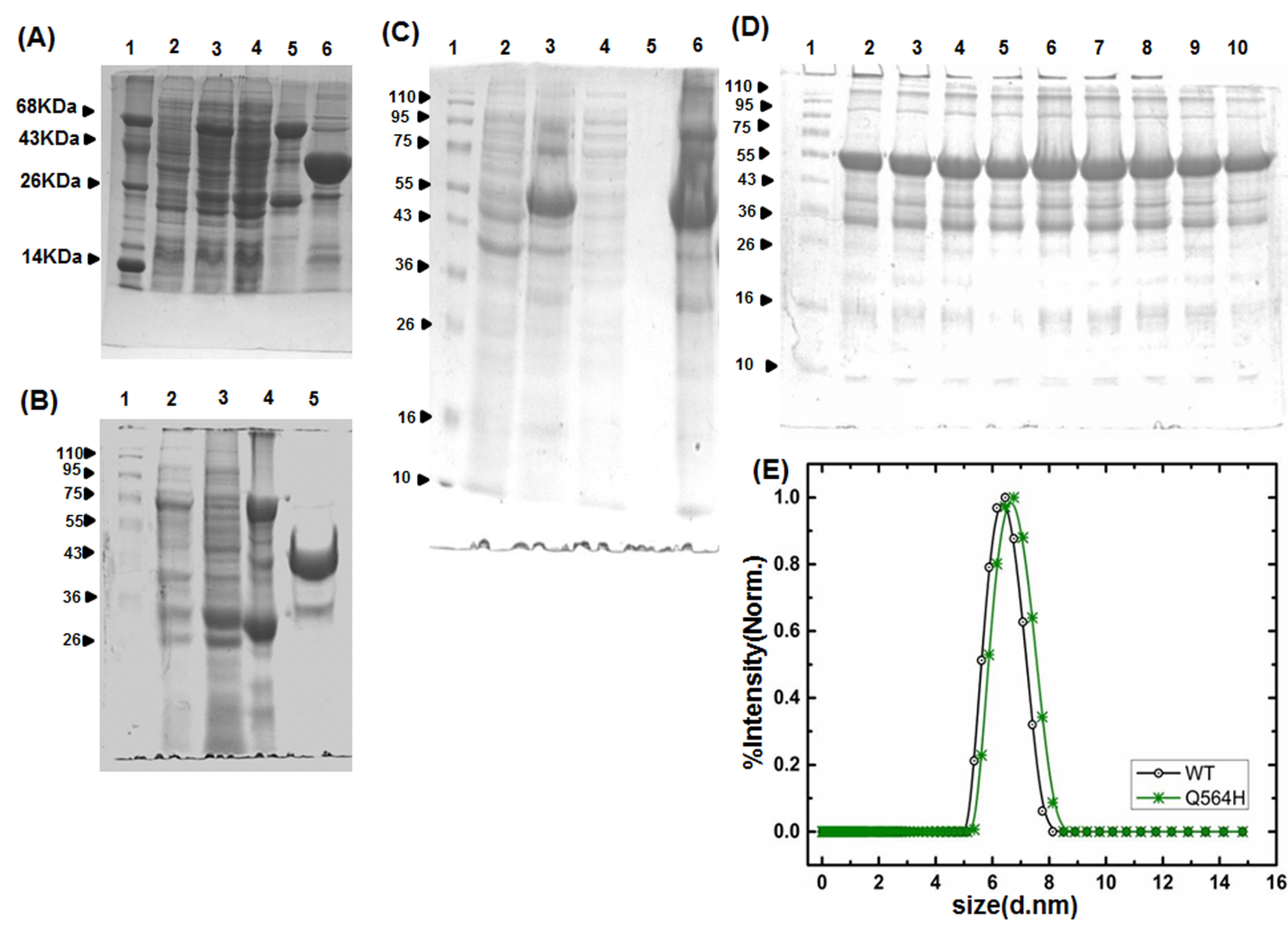


**Supplementary Figure 2: -** **(A)** Size exclusion chromatogram (Superdex-75 column) shows elution of BARD1 ARD-BRCT eluting at monomeric size. **(B)** Size exclusion chromatogram (Superdex -200 column) showing elution of Gln 564 His mutant eluting at monomeric size **(C)** Elution profile of standard protein in superdex-75 column.


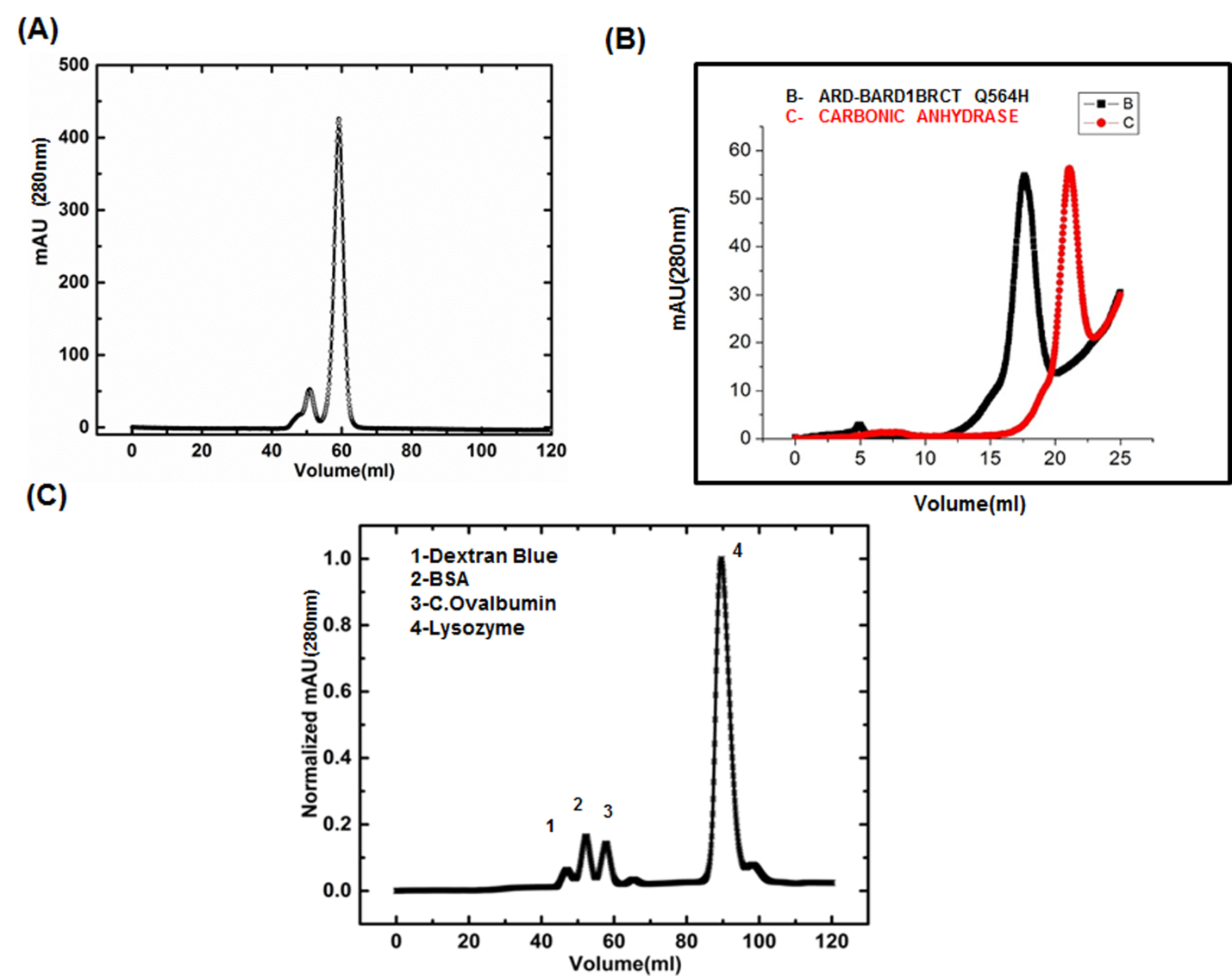


**Supplementary Figure 3:-** **(A and B)** Comparative glutaraldehyde cross-linking profile of BARD1 ARD-BRCT *wild-type* and Gln 564 His mutant protein. Lane M-Molecular weight marker and C-Untreated glutaraldehyde control. (**C and D)** Comparative limited trypsin proteolysis profile of BARD1 ARD-BRCT *wild-type*, Gln 564 His mutant protein. Lane M-Molecular weight marker and C-Untreated trypsin control

**
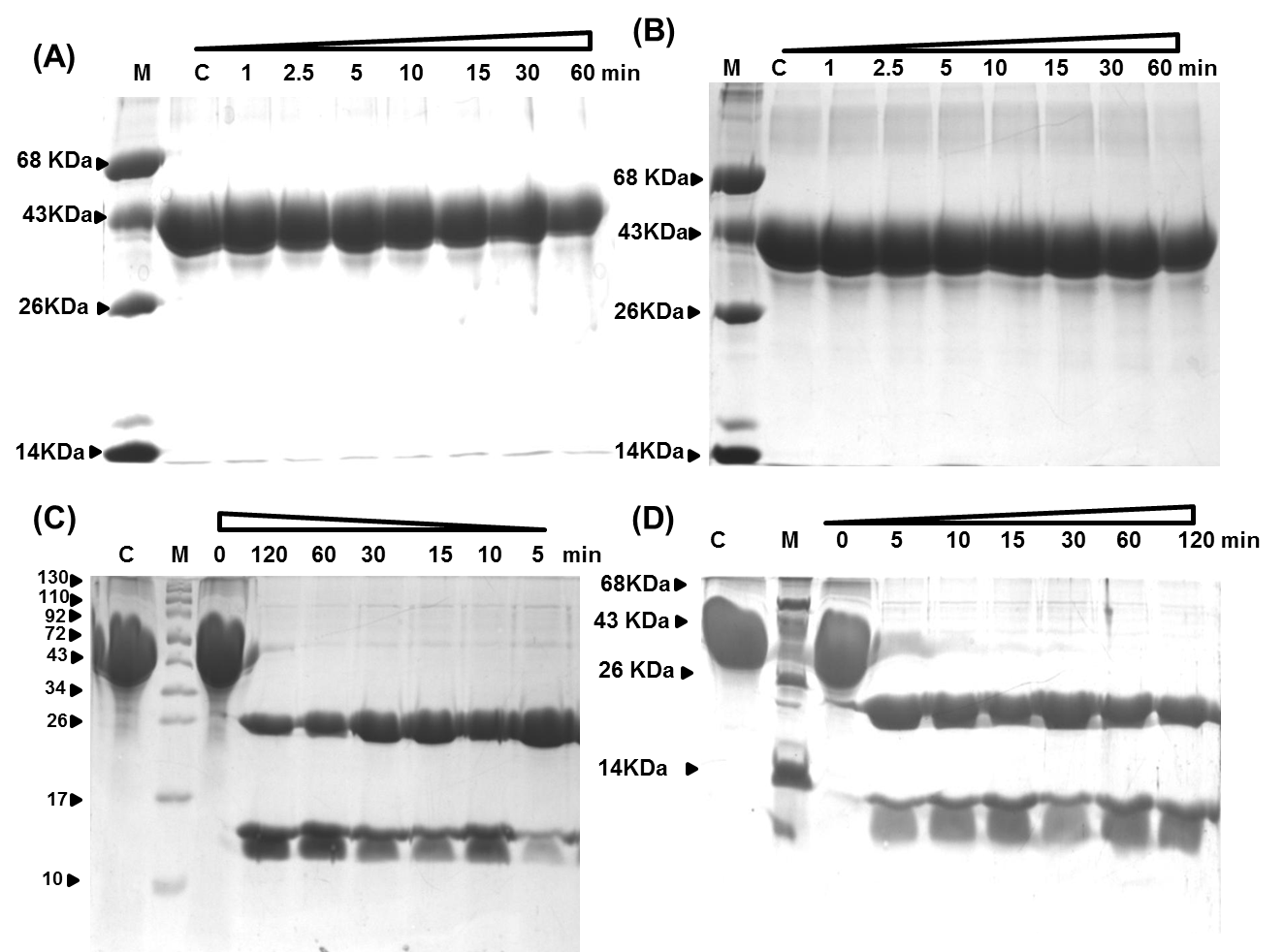
**

**Supplementary Figure 4:- (A and B)** Comparative CD spectra overlay of ARD-BARD1BRCT *wild-type* and mutant at 25°C (Blue) and 65°C (Red) respectively. **(C and D)** Average emission wavelength against GuHcl for ARD-BARD1BRCT *wild-type* and mutant protein respectively.


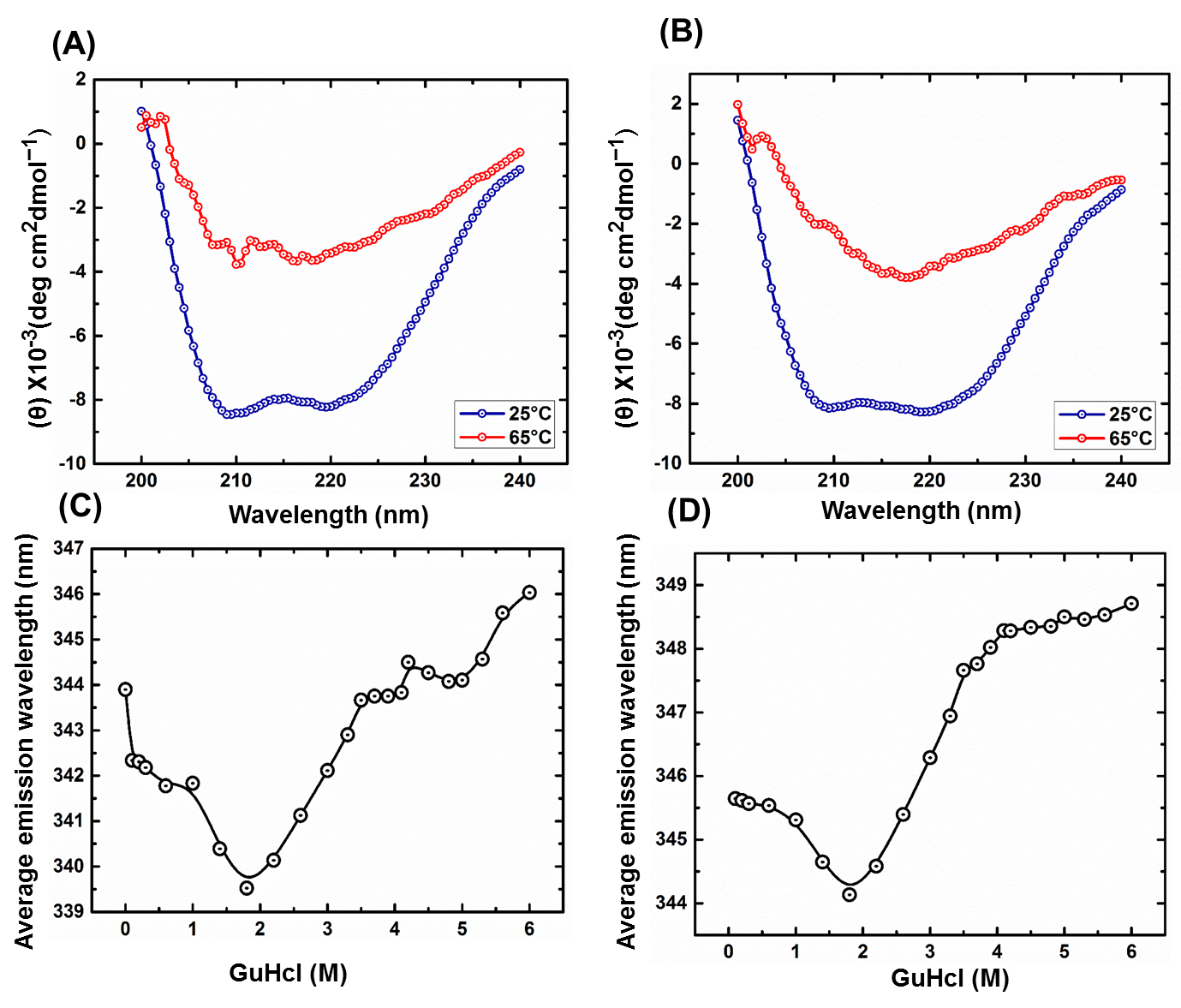


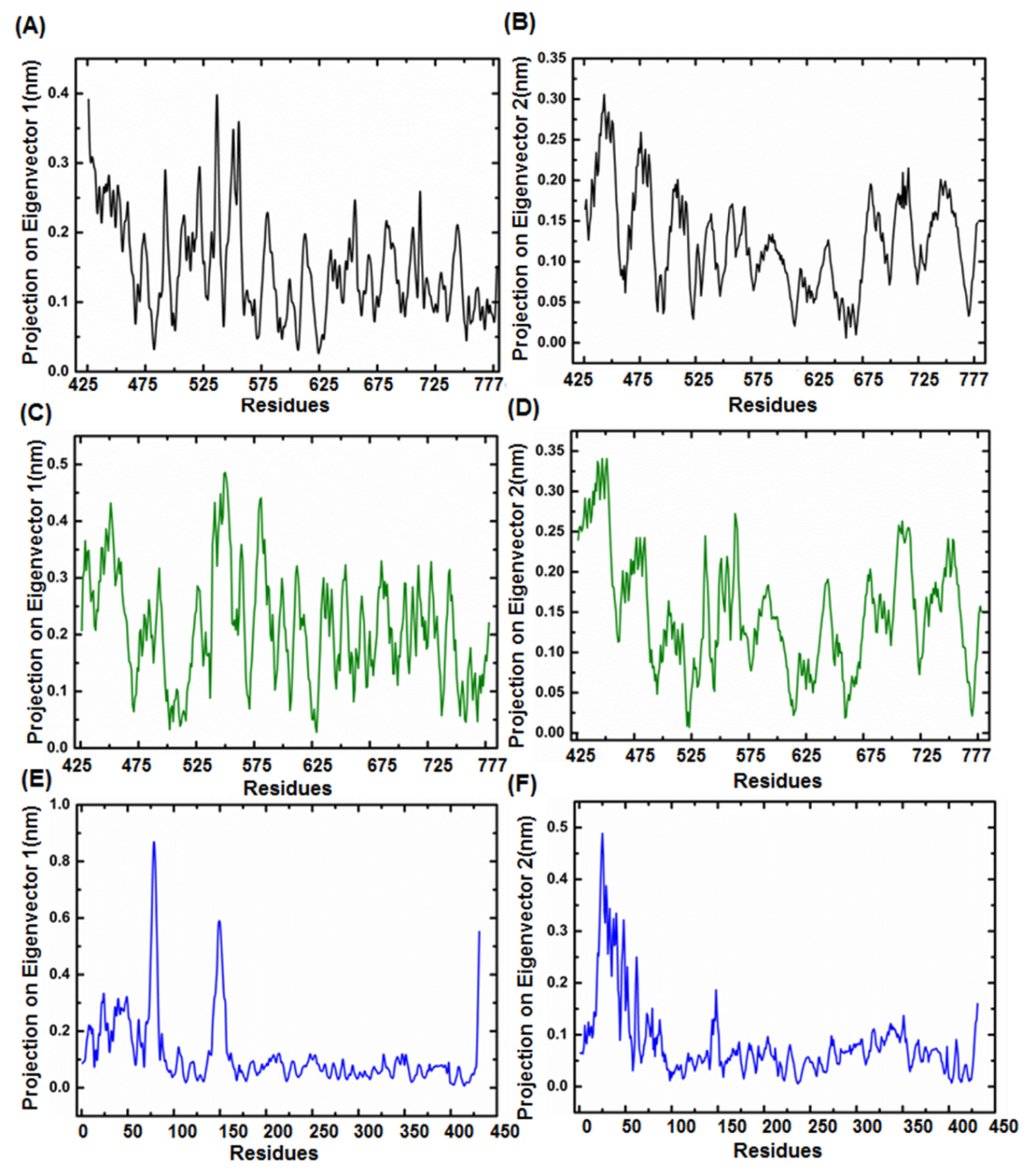
**Supplementary Figure 5: -** Comparative residual fluctuation analysis by plotting eigenvector 1 and 2 against residues for BARD1 ARD-BRCT *wild-type*, mutant and CstF50 **(A and B)** Comparative Eigenvector on residue projection profile of BARD1 ARD-BRCT *wild-type*, **(C and D)** Gln 564 His mutant protein, **(E and F)** CstF50 respectively.

**Supplementary Figure 6:-** **(A,B and C)** Free energy landscape of **(A)** BARD1 ARD-BRCT *wild-type* , **(B)** Gln 564 His mutant, **(C)** CstF50 respectively and **(D,E and F)** are representative structures of ARD1 ARD-BRCT *wild-type* , Gln 564 His mutant and CstF50 respectively.

**
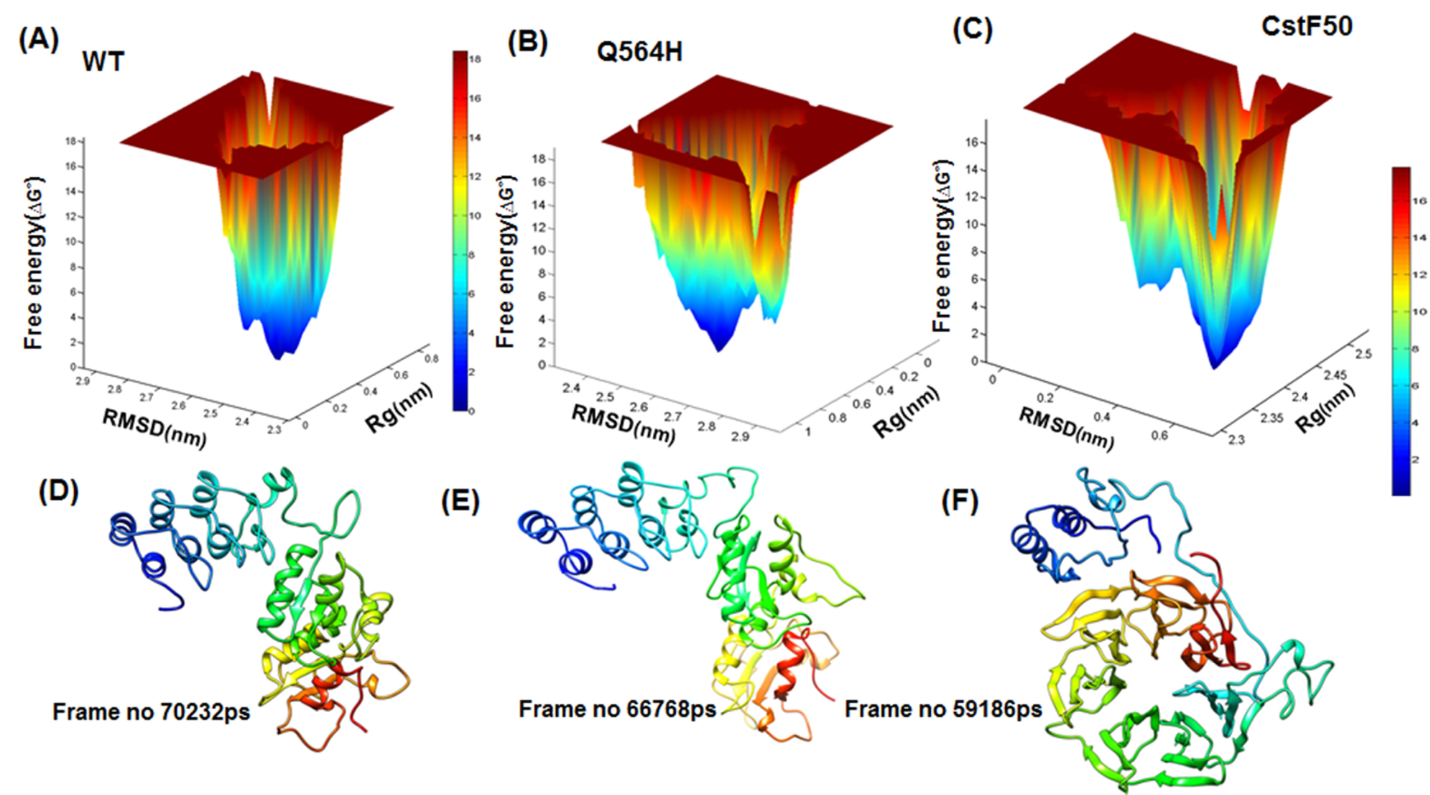
**

**Supplementary Figure 7:-** Inter-molecular interaction analysis of BARD1 ARD-BRCT *wild-type* - CstF50 and mutant complex. **Figure 7 A and B** shows hydrogen bonding interaction between BARD1 ARD-BRCT *wild-type* (Red)- CstF50 (Cyan), **Figure 7 C and D** shows non-hydrogen bonding interaction between mutant and CstF50 respectively. Chain-A is CstF50 (Cyan) and Chain-B is BARD1 ARD-BRCT Q564H mutant ((Red) respectively.


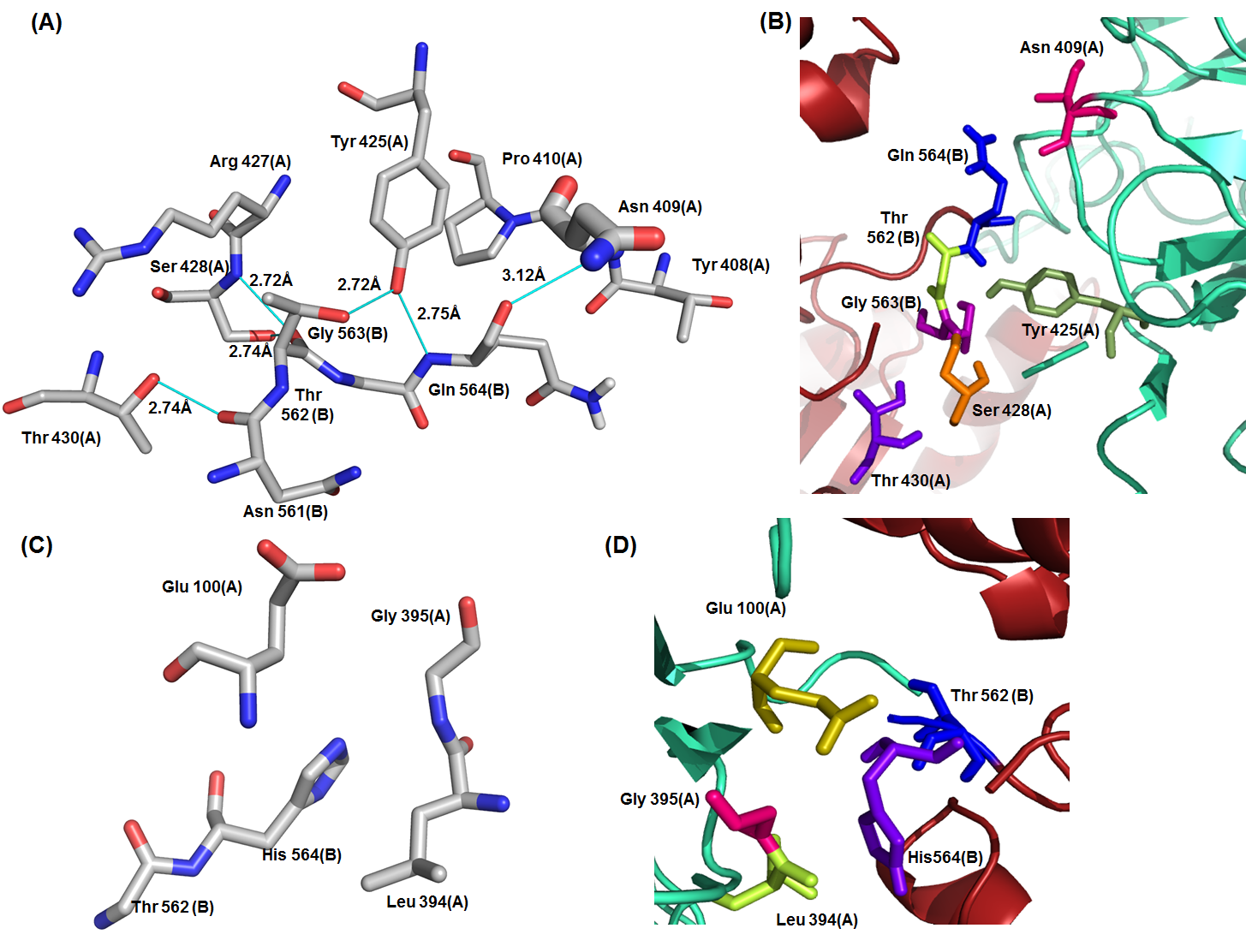

Supplement: Supplementary file 1 — Supplementary Dataset 1 [file 41598_2017_3816_MOESM1_ESM.doc]
